# Psychological Antecedents of Italian Sport Coaches’ Coaching Behaviors: The Role of Basic Psychological Needs, Motivation and Subjective Vitality

**DOI:** 10.3390/healthcare11202797

**Published:** 2023-10-22

**Authors:** Cristiano Inguglia, Sonia Ingoglia, Ignazio Leale, Nicolò Maria Iannello, Antonino Gennaro, David Manzano-Sánchez, Manuel Gómez-López, Giuseppe Battaglia

**Affiliations:** 1 Department of Psychology, Educational Science and Human Movement, University of Palermo, 90133 Palermo, Italy; cristiano.inguglia@unipa.it (C.I.); sonia.ingoglia@unipa.it (S.I.); nicolomaria.iannello@unipa.it (N.M.I.); giuseppe.battaglia@unipa.it (G.B.); 2 Laboratory of Behavioral Observation and Research on Human Development, Department of Psychology, Educational Science and Human Movement, University of Palermo, 90133 Palermo, Italy; 3 Sport and Exercise Sciences Research Unit, Department of Psychology, Educational Science and Human Movement, University of Palermo, 90133 Palermo, Italy; ignazio.leale@unipa.it; 4 Ph.D. Program in Health Promotion and Cognitive Sciences, University of Palermo, 90133 Palermo, Italy; 5 Regional Sports School of CONI Sicilia, 90141 Palermo, Italy; prof.gennaro@virgilio.it; 6 Department of Didactics of Musical, Plastic and Corporal Expression, Faculty of Psychology and Education, University of Extremadura, 06006 Badajoz, Spain; davidms@unex.es; 7 Department of Physical Activity and Sport, Faculty of Sports Sciences, University of Murcia, Santiago de la Ribera, 30720 Murcia, Spain; 8 Campus of International Excellence “Mare Nostrum”, University of Murcia, 30720 Murcia, Spain

**Keywords:** Self-Determination Theory, coaching style, sport coaches’ psychological well-being, motivation for coaching

## Abstract

The extant literature has identified some variables that are associated with sport coaches’ coaching style, like their basic psychological need satisfaction, their motivation for coaching, and their psychological well-being. Framed from a conceptual framework based on Self-Determination Theory applied to sport coaches, the current study explored how sport coaches’ basic psychological needs are associated with their motivation (intrinsic vs. external), subjective vitality, and coaching behaviors (autonomy-supporting vs. need-thwarting). Participants were 184 Italian sport coaches (males = 65%, M_age_ = 40.22, SD = 11.55, age range 19–74 years) from the “Centro CONI” project. They were requested to fill out online self-report questionnaires assessing the study variables. Structural equation modeling analyses suggested that sport coaches’ satisfaction of basic psychological needs was associated with higher levels of intrinsic motivation to train as well as with higher levels of vitality that, in turn, were associated with coaching behaviors supporting athletes’ autonomy. Moreover, sport coaches’ frustration of basic psychological needs was associated with higher levels of external motivation to train that, in turn, were associated with higher levels of a need-thwarting coaching style. Overall, the findings provided additional support for understanding how sport coaches’ basic psychological needs relate to their coaching styles.

## 1. Introduction

Several studies have adopted Self-Determination Theory [1,2] as a framework to explore psychological dynamics in a sport context [3,4,5]. These studies were mainly based on Basic Psychological Needs Theory [6], a sub-theory within SDT stating that people function most effectively in environments in which their basic psychological needs (namely, autonomy, competence, and relatedness) are supported and satisfied. Within this framework, authors have investigated the associations between athletes’ psychological need satisfaction, optimal psychological well-being, and performance in a number of sport activities [3,7,8,9,10] and in other fields, such as physical education classes [11,12]. For instance, Bartholomew et al. [3] highlighted that athletes’ experiences of need satisfaction during training were associated with high levels of well-being (i.e., positive affect), while their perceptions of need thwarting were associated with enhanced physiological stress, disordered eating behaviors, and depression.

Some authors have suggested that it is important to take into account the role of sport coaches in promoting athletes’ psychological well-being [4,5,6,7]. Indeed, a core assumption of BPNT is that significant others, such as sport coaches, play a key role in affecting athletes’ quality of experience in sport contexts via the nature of the social environment they set up [2,3]. In particular, sport coaches’ behaviors may create the conditions for both satisfying athletes’ sense of personal autonomy, competence, and relatedness (*need-supportive style*) and for frustrating their basic psychological needs with coercive, pressuring, and authoritarian behaviors (*need-thwarting or controlling style*) [3,13,14].

There is empirical evidence that sport coaches may affect athletes’ performances and psychological functioning through their interpersonal and coaching behaviors [4,15]. For instance, research has shown that sport coaches who engage in need-supportive behaviors are likely to satisfy athletes’ basic psychological needs, whereas those who adopt need-thwarting behaviors are likely to frustrate athletes’ basic psychological needs [14,15,16,17].

In light of these considerations, it is interesting to investigate the psychological antecedents of sport coaches’ interpersonal and coaching behaviors. The extant literature has identified some variables that are associated with sport coaches’ coaching style. First, sport coaches’ basic psychological need satisfaction has been revealed to be related to need-supportive coaching behaviors, while sport coaches’ basic psychological need frustration has been shown to be related to need-thwarting coaching behaviors [4,5]. Second, sport coaches’ motivation for coaching has been demonstrated to be associated with these coaching behaviors. In detail, intrinsic motivation is associated with sport coaches’ need-supportive behaviors, while extrinsic motivation is associated with need-thwarting behaviors [4,18,19]. Third, sport coaches’ psychological well-being (in terms of subjective vitality or positive affect) has been shown to be also related to their coaching behaviors, with higher levels of psychological well-being associated with need-supportive behaviors and lower levels of psychological well-being associated with need-thwarting behaviors [4,5].

### 1.1. Sport Coaches’ Basic Psychological Needs and Their Coaching Behaviors

The quality of sport coaches’ coaching behaviors has been demonstrated to be related to their satisfaction and frustration of basic psychological needs in the work context. In line with SDT [1,2], when individuals feel that work contexts support their basic psychological needs, they are more likely to experience need satisfaction and to enact need-supportive behaviors with other people. For instance, when sport coaches feel free to express their ideas and opinions (autonomy), learn new skills (competence), and like the people with whom they work (relatedness), it is more likely that they will perform need-supportive behaviors with the athletes they train [4,20,21]. On the contrary, when sport coaches perceive that their work context thwarts their basic psychological needs, they experience need frustration and, consequently, they are inclined to use need-thwarting interpersonal behaviors with their athletes [3,4,22]. Thus, when sport coaches feel pushed to behave in certain ways, disliked by other people, and incapable in some coaching situations, they can perceive high levels of need frustration. In turn, need frustration is related to coaching behaviors frustrating the basic psychological needs of athletes [4].

### 1.2. Sport Coaches’ Motivation for Coaching and Basic Psychological Need Satisfaction

The types of sport coaches’ coaching behaviors described so far have also been found to be related to motivation for coaching [23,24]. Indeed, in the framework of SDT, the quality of motivation is important to understand individual psychological functioning and interpersonal behaviors [6].

Specifically, this theory identifies three kinds of motivation that are expected to lead to different outcomes: amotivation, extrinsic motivation, and intrinsic motivation [6]. According to Deci and Ryan [1], these three types of motivation are placed along a continuum according to the extent to which the motivation for one’s behavior emanates from one’s self. *Amotivation* is the state of lacking an intention to act. *Extrinsic motivation* refers to behaviors performed in order to attain some separable outcome. Finally, intrinsic regulation is characterized by performing an activity for its inherent satisfaction, that is, performing an activity for the pleasure of the activity itself [1].

The quality of motivation for coaching has been shown to be associated with sport coaches’ need satisfaction or frustration [25]. When sport coaches work in contexts where they feel that their needs for autonomy, competence and relatedness are supported, then they are likely to experience an intrinsic pleasure in performing their work that leads them to engage in need-supportive behaviors with their athletes. Instead, when sport coaches perceive that their basic psychological needs are frustrated, they are more likely to experience extrinsic motivation for coaching and, thus, to engage in need-thwarting behaviors with their athletes [4,15,26].

Moreover, there is empirical evidence that motivation is associated with psychological well-being [27,28,29]. In particular, psychological well-being (i.e., in terms of sense of vitality or self-esteem) can be a consequence of intrinsic motivation, but, at the same time, it can promote this type of motivation. For instance, some authors have underlined that increased subjective well-being is associated with intrinsic motivation [27,29,30] and that low levels of psychological well-being are likely to be associated with extrinsic motivation [31].

### 1.3. Sport Coaches’ Psychological Well-Being, Basic Psychological Need Satisfaction, and Coaching Style

In the framework of SDT, sport coaches’ psychological well-being is also crucial to understand their behaviors towards their athletes. According to some authors [5,26], when sport coaches feel psychologically well, they are likely to function optimally in their coaching roles. In particular, Ryan et al. [32,33] proposed an eudaimonic definition of psychological well-being that occurs when individuals feel an integrated sense of self and realize their potential in terms of optimal psychological growth. To encompass this definition of well-being, Ryan and Frederick [33] developed the construct of *subjective vitality* that refers to a state of high positive energy emanating from the self.

In line with BPNT predictions, several studies have provided empirical evidence that the fulfillment of basic psychological needs is associated with people’s subjective vitality in sport context [7,13]. It is to be noted, however, that most of the extant research in this area has been conducted with athlete or student populations. Only a few studies [4,5] have found that sport coaches’ basic psychological needs were positively associated with their psychological well-being in terms of subjective vitality. For instance, Stebbing et al. [5] have revealed that the satisfaction of sport coaches’ basic psychological needs for autonomy and competence, but not relatedness, positively predicted their psychological well-being in terms of subjective vitality and positive affect. However, these findings need to be replicated in other countries and with other samples of sport coaches in order to determine whether they are generalizable.

Moreover, sport coaches’ subjective vitality has been shown to be related to their coaching style. Sport coaches who feel high positive energy emanating from themselves are likely to behave in a more positive way with their athletes, supporting their autonomy, competence, and relatedness needs. Instead, when sport coaches feel negative affect, they are likely to display need-thwarting behaviors towards their athletes. Although the relations between teachers’ psychological well-being and their behaviors towards students have been explored [34], only a few studies have investigated these topics in sport contexts. For instance, Stebbing et al. [5] showed that sport coaches’ subjective vitality was positively associated with need-supportive behaviors toward their athletes and negatively associated with need-thwarting behaviors. According to these authors, sport coaches’ subjective vitality may also play a mediating role in the association between sport coaches’ basic psychological needs and their coaching style. However, further studies are needed to corroborate these findings.

Currently, it seems that few studies have analyzed all these psychological antecedents of sport coaches’ interpersonal behaviors together [4,5] and that no studies like this have been carried out in Italy so far. Thus, the present study aimed at filling these gaps through analyzing the associations between Italian sport coaches’ satisfaction and frustration of basic psychological needs, their motivation, subjective vitality, and coaching behaviors.

### 1.4. The Current Study

The present study sought to contribute to a more detailed knowledge of the relations between sport coaches’ satisfaction and frustration of basic psychological needs, their motivation for coaching, their subjective vitality, and their coaching style. In doing so, this study also explored the mediating role of sport coaches’ motivation for coaching and subjective vitality in the association between sport coaches’ satisfaction and frustration of basic psychological needs and their coaching behaviors. To the best of our knowledge, no previous studies have examined a combination of such variables in a comprehensive model.

Framed using a conceptual framework based on an integration of SDT applied to sport contexts [3,5], the following hypotheses have been proposed:

**H1:**  *Sport coaches’ satisfaction of basic psychological needs is positively associated with a need-supportive coaching style, whereas sport coaches’ frustration of basic psychological needs is positively associated with a need-thwarting coaching style.*

**H2:**  *Sport coaches’ satisfaction of basic psychological needs is positively associated with intrinsic motivation for coaching, whereas sport coaches’ frustration of basic psychological needs is positively associated with extrinsic motivation for coaching.*

**H3:**  *Sport coaches’ satisfaction of basic psychological needs is positively associated with their subjective vitality, whereas sport coaches’ frustration of basic psychological needs is negatively associated with their subjective vitality.*

**H4:**  *Sport coaches’ satisfaction of basic psychological needs is indirectly associated with a need-supportive coaching style through the mediating role of both subjective vitality and motivation for coaching—the greater the need satisfaction, the higher the subjective vitality and intrinsic motivation, and the more teachers tend to report higher levels of a need-supportive coaching style.*

**H5:**  *Sport coaches’ frustration of basic psychological needs is indirectly associated with a need-supportive coaching style through the mediating role of both subjective vitality and motivation for coaching—the greater the need frustration, the lower the subjective vitality, the higher the extrinsic motivation, and the more teachers tend to report higher levels of a need-thwarting coaching style.*

**H6:**  *With regard to the relation between sport coaches’ subjective vitality and motivation for coaching, in line with research guided by SDT that has shown that higher levels of psychological well-being (i.e., subjective vitality or self-esteem) are associated with more self-determined motivation [27,29,30], it was expected that subjective vitality would have been positively related to intrinsic motivation and negatively related to extrinsic motivation.*

**Figure 1:**
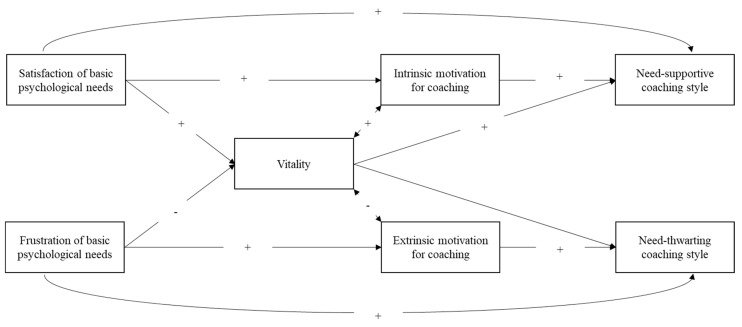
Hypothesized model. Age, gender, and years of coaching, their effects on study variables, and residuals are not reported for clarity purposes.

The hypothesized model is reported in Figure 1. Age, gender, and years of coaching were specified as covariates in the model.

## 2. Materials and Methods

### 2.1. Design

The study design was a survey, with confirmative goals, since it was aimed at testing a series of hypotheses originating in the context of the SDT.

### 2.2. Participants

The sample was recruited using a convenience sampling technique due to the geographical proximity and availability of the participants, who were required to train in a specific course at the time of the research. The sample was composed of 184 Italian sport coaches (males = 65%) living in Sicily (South of Italy), aged between 19 and 74 years old (M = 40.22, SD = 11.55). Other socio-demographic characteristics are reported in Table 1.

### 2.3. Procedure

All coaches completed the questionnaire anonymously in collective sessions, which took approximately 25 min, during the mandatory training course of the project “Centro CONI—Orientamento e Avviamento allo Sport”, organized by the Comitato Olimpico Nazionale Italiano*—*CONI in Sicily. The project promoted multiple sports during children’s developmental age, and trained CONI coaches about this topic, in Italy. The subject and mandatory nature of the course to obtain the CONI certification promoted coaches’ participation in the professional training. Although involvement in the current research was voluntary, all course coaches agreed.

All participants were informed about the objectives of the study and gave their informed consent. Data collection was conducted with an online questionnaire administered by researchers. Ethical approval for this study was obtained from Ethics Committee of University of Palermo (Approval number N. 67/2021).

### 2.4. Measures

*Satisfaction of basic psychological needs*. Participants were assessed using the positive items from the Basic Need Satisfaction at Work Scale (BNSAW) [32], which were adapted to the coaching context. In line with the modifications suggested by Ntoumanis (2005), only 12 items were used. Competence satisfaction was assessed using three items (e.g., “I have been able to learn interesting new skills when I coach”), autonomy satisfaction was assessed using four items (e.g., “I feel like I can make a lot of inputs to deciding how my coaching gets done”), and relatedness satisfaction was assessed using five items (e.g., “When I coach, people care about me.”). Participants were asked to indicate their degree of agreement or disagreement on a 7-point scale ranging from 1 (totally disagree) to 7 (totally agree). In the current study, the scale had adequate internal consistency, with Cronbach’s α equal to 0.92.

*Frustration of basic psychological needs*. Participants were assessed using the Psychological Need Thwarting Scale in Sport [20]. The scale consists of 12 items: four assessing competence frustration (e.g., “There are time when I am told things that make me feel incompetent”), four assessing autonomy frustration (e.g., “I feel pushed to behave in certain ways”), and four assessing relatedness frustration (e.g., “I feel other people dislike me”). Participants were asked to indicate their degree of agreement or disagreement using a 7-point scale ranging from 1 (totally disagree) to 7 (totally agree). In the current study, the scale had adequate internal consistency, with Cronbach’s α equal to 0.93.

*Motivation for coaching*. Participants were administered the Coach Motivation Questionnaire (CMQ) [35]. The CMQ is a 6-factor scale measuring sport motivation according to each of the types of behavioral regulation according to SDT (motivation, external regulation, introjected regulation, identified regulation, integrated regulation, and intrinsic motivation). For the purposes of the current study, only two subscales have been taken into account: external regulation, which evaluates extrinsic motivation, namely motivation regulated by compliance, conformity, and external rewards and punishments; and intrinsic regulation, which evaluates intrinsic motivation, namely when an individual is driven by interest, enjoyment, and the satisfaction inherent in the activity he or she is engaging in. They are both composed of 4 items (e.g., “The reason why I am coaching is because I like extrinsic rewards, i.e., money”; “The reason why I am coaching is because I get a good feeling out of it”). Items are rated on a 7-point Likert scale, ranging from 1 (totally disagree) to 7 (totally agree). In the current study, the subscales had adequate internal consistency, with Cronbach’s α equal to 0.76 for intrinsic regulation, and to 0.84 for external regulation.

*Subjective Vitality*. Participants were assessed using the 7-item Subjective Vitality Scale [33]. It assessed the degree to which participants felt psychologically vigorous and energized while coaching during the last month. Items are preceded with the stem “When I am coaching…” (e.g., “When I am coaching, I feel alive and vital”). Items are rated on a 7-point Likert scale, ranging from 1 (totally disagree) to 7 (totally agree). In the current study, the scale had adequate internal consistency, with Cronbach’s α equal to 0.79.

*Coaching style*. Participants completed the 24-item Interpersonal Behaviors Questionnaire (IBQ-Self) scale [24,36]. It assessed the extent to which they believed they used six types of interpersonal behaviors (four items per subscale) introduced with the stem “When I am with my athletes”: autonomy-supportive (AS; e.g., “I give them the freedom to make their own choices”), autonomy-thwarting (AT; e.g., “I impose my opinions on them”), competence-supportive (CS; e.g., “I provide them valuable feedback”), competence-thwarting (CT; e.g., “I point out that they will likely fail”), relatedness-supportive (RS; e.g., “I am interested in what they do.”), and relatedness-thwarting (RT; e.g., “I do not comfort them when they are feeling low”). Items are rated on a 7-point Likert scale, ranging from 1 (totally disagree) to 7 (totally agree). In the current study, two total scores were calculated, one for the need-supportive coaching style (AS, CS, RS) and one for the need-thwarting (AT, CT, RT) coaching style. The subscales had adequate internal consistency, with Cronbach’s α equal to 0.86 for the need-supportive coaching style and 0.84 for the need-thwarting coaching style.

### 2.5. Data Analysis

Preliminarily, we computed univariate descriptive statistics (mean and standard deviation) of the study variables. We also computed Pearson correlation coefficients in order to examine their interrelations. Analyses were performed using the jamovi project’s (2023) Jamovi computer software (version 2.4), retrieved from https://www.jamovi.org, accessed 30 September 2023.

Then, we ran a path analysis in order to test the hypothesized model. As a first step, we ran a model in which no mediators were specified, and the satisfaction and frustration of basic psychological needs had only direct effects on need-supportive and need-thwarting coaching styles. The results of this model are reported in the Supplementary Materials. To test the hypothesized model, Preacher and Hayes’s bootstrapping approach was used; aligned with previous recommendations [36,37,38], confidence intervals of the direct and indirect effects with 5000 bootstrap replication samples were used, and a bias-corrected 95% CI was applied. The maximum likelihood (ML) estimation method was used. To statistically evaluate the closeness of the hypothetical model to the empirical data, multiple goodness-of-fit indexes were used, including the Comparative Fit Index (CFI), the Root Mean Square Error of Approximation (RMSEA), and the Standardized Root Mean Square Residual (SRMR). The chi-square test of model fit was not used as an evaluation of absolute fit because of its sensitivity to sample size. CFI values ≥ 0.90 and RMSEA and SRMR values ≤ 0.08 were interpreted as evidence of acceptable fit to the data, while CFI values ≥ 0.95 and RMSEA and SRMR values ≤ 0.05 were interpreted as evidence of excellent fit to the data [39,40]. Analyses were performed using Mplus 7 [41].

## 3. Results

### 3.1. Descriptive Statistics and Correlation

Descriptive statistics of the study variables and Pearson correlation coefficients are reported in Table 2. The satisfaction and frustration of basic psychological needs were not correlated with each other; basic psychological need satisfaction was positively and significantly correlated with intrinsic and extrinsic motivation for coaching, subjective vitality, and a need-supportive coaching style; basic psychological need frustration was positively and significantly correlated with extrinsic motivation for coaching and a need-thwarting coaching style. Intrinsic and extrinsic motivation were not correlated with each other; intrinsic motivation was positively and significantly correlated with a need-supportive coaching style and subjective vitality; extrinsic motivation was positively and significantly correlated with a need-thwarting coaching style. Gender, age, and years of coaching were not significantly correlated with the study variables, with the only exception of years of coaching being positively correlated with a need-supportive coaching style.

### 3.2. The Hypothesized Model

**Figure 2:**
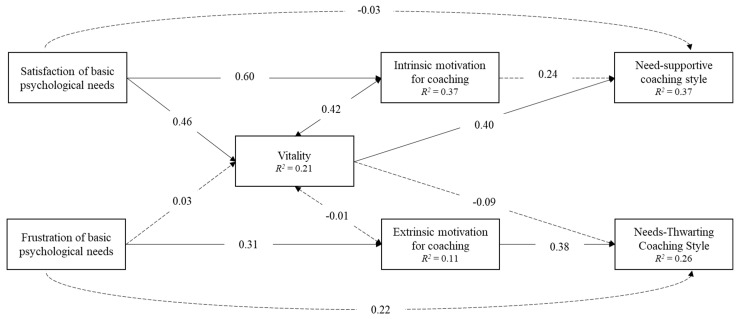
Standardized solution of the statistical model. Age, gender, years of coaching, their effects on study variables, and residuals are not reported for clarity purposes. All estimates are significant at *p* < 0.05 with the exception of those represented by dashed lines.

Gender, age, and years of coaching were included as control variables. The model had a good fit to the data; χ^2^ (13) = 32.92, *p* = 0.002, CFI = 0.939, RMSEA = 0.091 [90% CI: 0.05, 0.10], and SRMR = 0.050. The standardized solution of the model is reported in Figure 2; the unstandardized path estimates, standard errors, and 95% confidence intervals of direct and indirect effects are reported in Table 3.

Differently than hypothesized, sport coaches’ satisfaction of basic psychological needs had no significant direct effect on need-supportive coaching style, and sport coaches’ frustration of basic psychological needs had no significant direct effect on need-thwarting coaching style.

As hypothesized, sport coaches’ satisfaction of basic psychological needs was positively associated with intrinsic motivation for coaching, and sport coaches’ frustration of basic psychological needs was positively associated with extrinsic motivation for coaching.

As hypothesized, sport coaches’ satisfaction of basic psychological needs was positively associated with their psychological subjective vitality, but differently than hypothesized, sport coaches’ frustration of basic psychological needs had no significant direct effect on subjective vitality.

As hypothesized, subjective vitality was positively correlated with intrinsic motivation, but differently than hypothesized, it was not significantly correlated with extrinsic motivation.

As hypothesized, sport coaches’ satisfaction of basic psychological needs was indirectly associated with need-supportive coaching style through the mediating role of subjective vitality (β = 0.18, *p* = 0.001, 90% CI: 0.09, 0.28) and the mediating role of intrinsic motivation for coaching (β = 0.15, *p* = 0.039, 90% CI: 0.03, 0.26).

As hypothesized, sport coaches’ frustration of basic psychological needs was indirectly associated with need-supportive coaching style through the mediating role of extrinsic motivation for coaching (β = 0.12, *p* < 0.001, 90% CI: 0.07, 0.17).

## 4. Discussion

Grounded within the SDT framework applied to sport contexts [3,5], the current study investigated some psychological antecedents of Italian sport coaches’ coaching behaviors. In particular, the associations between sport coaches’ satisfaction and frustration of basic psychological needs, their motivation for coaching, their subjective vitality, and their coaching style have been analyzed. The findings of the study partially confirmed the initial hypotheses.

With regard to H1, the findings are not in line with the predictions since a statistically significant association was not found between either sport coaches’ basic psychological need satisfaction and need-supportive coaching style nor between sport coaches’ basic psychological need frustration and need-thwarting coaching style. However, the simple correlations (see Table 2) between these variables are significant and align with the hypotheses. Thus, it could be possible that the strength of these associations becomes weaker when they are considered in a more complex model together where other variables mediate the relations between sport coaches’ basic psychological need satisfaction/frustration and their coaching styles. The mediating role of these variables is discussed below.

H2 was fully confirmed. Accordingly with the initial prediction, when sport coaches feel that their basic psychological needs are satisfied in their work context, they are more intrinsically motivated to coach, and they are pushed to coach by the pleasure derived from the coaching itself. Instead, when sport coaches feel that their psychological basic needs are frustrated in their work context, they are moved by extrinsic motives, and they train just to earn external rewards or to obtain recognition from other people. These findings are in line with the literature on these topics in educational and sport contexts that underline an association between the satisfaction or frustration of teachers or sport coaches’ basic psychological needs and their autonomous or controlled motivation to teach or coach [3,18,42,43]. Particularly, intrinsic motivation may be linked to the satisfaction of sport coaches’ basic psychological needs in the work context. Therefore, sport coaches who feel autonomous, competent, and connected with others in performing their own work are also pleased to engage in coaching activity just for the gratification that comes from enriching athletes with new insights and knowledge. On the contrary, if the work context undermines sport coaches’ basic psychological needs, this may result in need frustration, leading to a controlled or extrinsic motivation to coach [15,23].

Moreover, H3 was only partially confirmed since sport coaches’ basic psychological need satisfaction was positively and significantly associated with their subjective vitality, whereas sport coaches’ basic psychological need frustration was not significantly associated with their subjective vitality. These findings provide further evidence that need satisfaction and need frustration are independent constructs that may be associated with different outcomes [3,44,45]. Also, Costa et al. [45] have found similar results inferring that while need satisfaction is essential for flourishing and, consequently, is linked to positive outcomes like subjective vitality, need frustration is more likely to be associated with negative outcomes, which are not considered in the current study. Thus, the absence of this significant association could be explained in light of these considerations.

H4 and H5 were almost fully confirmed since most of the predicted associations were statistically significant. In the tested model, the association between sport coaches’ basic psychological need satisfaction and a need-supportive coaching style was significantly mediated by subjective vitality, but not by the intrinsic motivation for coaching, even if the latter was positively correlated with subjective vitality. In line with other studies [5,46], when sport coaches feel that their basic psychological needs are supported by their work context, they tend to experience high positive energy emanating from the self and, consequently, they tend to provide athletes with need-supportive strategies that may endorse their sense of choice, responsibility, and engagement. At the same time, need satisfaction is also positively associated with sport coaches’ intrinsic motivation that, in turn, is positively related to their subjective vitality but not to their need-supportive coaching style. Instead, the association between sport coaches’ basic psychological need frustration and a need-thwarting coaching style was significantly mediated only by extrinsic motivation for coaching and not by subjective vitality. As discussed above, in the hypothesized model, need frustration seems to play a different role from need satisfaction since it is not related to psychological well-being in terms of subjective vitality, but it is linked to extrinsic motivation and, indirectly, to a need-thwarting coaching style. In line with Rocchi [47], when sport coaches feel that their basic psychological needs are thwarted in their work context, they are likely to show higher levels of controlled motivation and to work just for external rewards (e.g., money). This results in a coaching style that thwarts athletes’ needs for autonomy (i.e., imposing their ideas or using intimidating language), competence (i.e., emphasizing their faults or mistakes), and relatedness (i.e., tending to isolate them) [13,16,42].

Finally, our predictions about the relations between sport coaches’ subjective vitality and motivation for coaching (H6) were partially confirmed since sport coaches who reported higher levels of subjective vitality tended to also show higher levels of intrinsic motivation to coach, whereas, differently from what we hypothesized, lower levels of subjective vitality were not significantly related to an extrinsic motivation to coach. With regard to the first prediction, empirical evidence shows that those sport coaches who are intrinsically motivated towards coaching tend to feel active, vital, and full of energy, and this is congruent with previous research framed within SDT [23,33]. With regard to the second prediction, the findings of the study highlight, once again, that in the hypothesized model, controlled motivation seems to be associated only with need frustration and need-thwarting behaviors. This is in line with similar research that did not find an association between coaches’ controlled motivation and their sense of subjective vitality [23].

### Limitations and Future Directions

The current study has potential limitations that must be taken into consideration. First, the cross-sectional design of the study makes it difficult to clarify the direction of the relations among the study variables within the model. Hence, further longitudinal studies are needed to establish the direction of the relations among these variables. However, the current study was based on a strong theoretical framework, which helped shed some light on possible antecedents of sport coaches’ interpersonal and coaching behaviors.

Second, the number of participants is quite limited, and they are only from Sicily. Thus, the sample is not representative of all sport coaches in Italy, and the conclusions cannot be generalized. Future research should try to involve a larger sample of sport coaches, including those who live in other Italian regions.

Third, as in other studies, the current work used self-reported measures, which could be affected by social desirability bias, especially with regard to the motivation to coach and coaching styles. Although, in the main study, a social desirability measure was included, it would be better that future studies use objective assessments of sport coaches’ behavior (e.g., independent observations).

## 5. Conclusions

Despite these shortcomings, the findings of the present study may contribute to advancing the current literature in several ways. First, to the best of our knowledge, no previous research has examined the relations between sport coaches’ basic psychological need satisfaction/frustration and coaching styles in Italy. Second, there are no studies that investigate the associations among sport coaches’ basic psychological need satisfaction/frustration, motivation for coaching, subjective vitality, and coaching styles in a comprehensive model. Thus, the current study provides further empirical evidence for understanding the mechanisms behind sport coaches’ coaching styles.

Taken together, the findings of this study seem to highlight that there are two different pathways leading from need satisfaction/frustration to coaching styles. The first one involves sport coaches’ basic psychological need satisfaction, subjective vitality, and need-supportive coaching styles. In this sense, the experience of working in a context in which sport coaches’ basic psychological needs are met leads them to feel vital and full of energy and, in turn, to display need-supportive behaviors towards their athletes. In this pathway, intrinsic motivation also plays a role, but the main mediator is subjective vitality. Instead, the second pathway involves sport coaches’ basic psychological need frustration, extrinsic motivation, and need-thwarting coaching styles. In particular, the frustration of sport coaches’ basic psychological needs is associated with higher levels of controlled motivation that, in turn, are associated with need-thwarting coaching styles. In this pathway, subjective vitality does not play a mediating role.

Finally, the findings of this study have practical implications for sport coaches’ work and training, as well as for athletes’ psychological well-being. In line with the European Commission and the Council of Europe’s increasing recognition of the potential contribution of sport coaching to the well-being of athletes (Council of the European Union, 2020; European Commission, 2020), sport coaches are requested to move beyond their traditional role (i.e., developing sporting abilities) towards being mentors and educators [48]. To achieve this goal, research-based knowledge to plan and implement sport coaches’ development and education is needed.

In light of the current results, sport clubs, sport associations, gyms, and all the other contexts in which sport coaches work should pay attention to create a supportive environment for sport coaches’ basic psychological need satisfaction to promote their sense of well-being and need-supportive coaching styles. At the same time, such contexts should avoid need frustration to prevent extrinsic motivation and need-thwarting coaching styles. Following the recommendations developed in the framework of the PEAK (Policy, Evidence, and Knowledge in Coaching) project [49], more support for sport coaches is needed through the clear definition of their role in the organizations in which they work, the provision of comprehensive employment contracts, the involvement of under-represented groups in sport coaching (e.g., based on sex, ethnicity, sexual orientation, class, ability status, etc.), and the establishment of a positive relational climate.

Moreover, these results should be useful for designing educational programs addressed to sport coaches. Such programs raise sport coaches’ awareness of the effects that their coaching styles may have on their athletes’ basic psychological needs, and, consequently, on their athletes’ psychological well-being and performance. At the same time, educational programs for sport coaches should be aimed at equipping them with methods and techniques to support the satisfaction of athletes’ basic psychological needs (e.g., providing athletes with opportunities for initiative-taking and independent work, providing athletes with choices within specific rules and limits). One interesting example of the implementation of the SDT framework in a sport coach development program is the Motivation Activation Program in Sports (MAPS [50]), which was developed to add an interpersonal-style perspective for coaches in the Norwegian Ski Federation’s educational system.

## Figures and Tables

**Table 1:** Socio-demographic characteristics of participants.

	*Mean* (*SD*) or %
*Coaching years*	11.79 (10.35)
*Marital status*	
Single	39%
Married or cohabiting	55%
Divorced/separated	5%
Widower/widow	1%
*Education*	
Middle school	3%
High school	44%
Degree	41%
Post-degree	12%
*Sports degree*	
No degree	13%
Technical qualification sports operator	52%
Bachelor degree	10%
Master degree	25%
*Trained category*	
Amateur	23%
Non-professional	65%
Professional	11%
*Awards as a coach*	
Yes	41%
No	59%
*Age group of people trained*	
Children	36%
Adolescents	55%
Adults	9%
*Coach as main occupation*	
Yes	40%
No	60%

**Table 2:** Mean (*M*) standard deviation (*SD*) values and Pearson correlation coefficients of the study variables.

	*M*	*SD*	1	2	3	4	5	6	7	8	9	10
1 Satisfaction of basic psychological needs	5.84	1.09	-									
2 Frustration of basic psychological needs	1.85	1.15	−0.09	-								
3 Intrinsic motivation for coaching	6.5	0.95	0.60 ***	−0.11	-							
4 External motivation for coaching	3.16	1.97	0.27 ***	0.30 ***	0.09	-						
5 Need-supportive coaching style	6.03	0.84	0.32 ***	−0.09	0.48 ***	−0.02	-					
6 Need-thwarting coaching style	2.4	0.96	0.06	0.34 ***	−0.04	0.43 ***	−0.04	-				
7 Subjective vitality	5.63	1.01	0.47 ***	−0.04	0.58 ***	0.09	0.56 ***	−0.09	-			
8 Gender	1.33	0.47	0.01	0.02	0.07	−0.05	0.12	−0.10	0.12	-		
9 Age	40.22	11.55	0.01	0.10	0.07	−0.07	0.03	0.07	0.02	−0.13	-	
10 Years of coaching	11.79	10.35	0.11	0.08	0.12	−0.02	0.21 **	−0.01	0.14	−0.01	0.66 ***	-

*Note*. Gender was coded as male = 1 and female = 2. ** *p* ≤ 0.01. *** *p* ≤ 0.001.

**Table 3:** Unstandardized path estimates for direct and indirect effects, SEs, and 95% CIs for the hypothesized model.

	*b*	*SE*	Bias-Corrected 95% CI
*Direct effect*			
Satisfaction of basic psychological needs → Need-supportive coaching style	−0.02	0.07	[−0.15, 0.13]
Satisfaction of basic psychological needs → Intrinsic motivation for coaching	0.52	0.12	[0.29, 0.74]
Satisfaction of basic psychological needs → Vitality	0.42	0.11	[0.21, 0.63]
Frustration of basic psychological needs → Need-thwarting coaching style	0.18	0.11	[−0.01, 0.40]
Frustration of basic psychological needs → Extrinsic motivation for coaching	0.53	0.13	[0.25, 0.77]
Frustration of basic psychological needs → Vitality	0.02	0.06	[−0.10, 0.13]
Vitality → Need-supportive coaching style	0.34	0.09	[0.15, 0.51]
Vitality → Need-thwarting coaching style	−0.09	0.06	[−0.21, 0.04]
Intrinsic motivation for coaching → Need-supportive coaching style	0.22	0.12	[0.01, 0.46]
Extrinsic motivation for coaching → Need-thwarting coaching style	0.18	0.04	[0.11, 0.28]
*Indirect effect* via *vitality*			
Satisfaction of basic psychological needs → Need-supportive coaching style	0.14	0.05	[0.06, 0.26]
Frustration of basic psychological needs → Need-thwarting coaching style	−0.01	0.01	[−0.02, 0.01]
*Indirect effect* via *intrinsic motivation*			
Satisfaction of basic psychological needs → Need-supportive coaching style	0.11	0.06	[0.01, 0.24]
*Indirect effect* via *extrinsic motivation*			
Frustration of basic psychological needs → Need-thwarting coaching style	0.10	0.03	[0.05, 0.16]

## Data Availability

The data presented in this study are available from the corresponding author upon request.

## Supplementary Materials

The following supporting information can be downloaded at: https://www.mdpi.com/article/10.3390/healthcare11202797/s1, Figure S1. Model with no mediators.
