# Supplementary material for: Psychological Antecedents of Italian Sport Coaches’ Coaching Behaviors: The Role of Basic Psychological Needs, Motivation and Subjective Vitality
Source: Healthcare (Basel). 2023 Oct 22;11(20):2797. doi: 10.3390/healthcare11202797 (PMC10606446; doi:10.3390/healthcare11202797)
Supplement: Supplementary file 1 [file healthcare-11-02797-s001.zip › healthcare-2667008-supplementary.pdf]

3.1S. Model with no mediators

We tested a model in which no mediators were specified, and in which satisfaction and frustration of basic psychological needs had only direct effects on need-supportive and need-thwarting coaching style. The model had a good fit to the data, χ^2 (10) = 8.61, p = .57, CFI = 1.00, RMSEA = 0.00 [90% CI: .00, .07], SRMR = .037. The standardized solution of the model is reported in Figure 1S. Results showed that need-supportive coaching style was positively and significantly related with satisfaction of basic psychological needs and years of coaching; moreover, need-thwarting coaching style was positively and significantly related with frustration of needs.

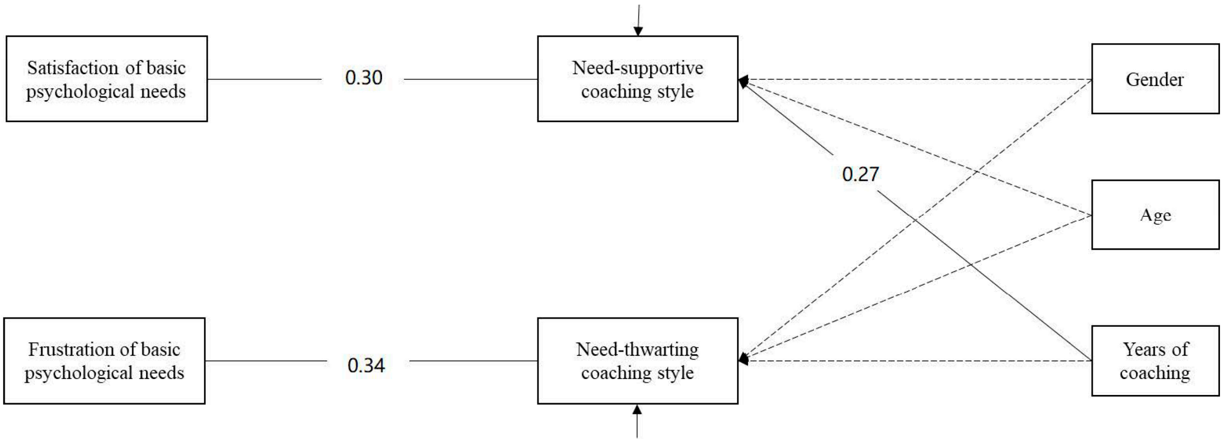

Figure S1. Model with no mediators.
